# Management of Uremic Xerosis and Chronic Kidney Disease (CKD)-Associated Pruritus (CKD-ap) With Topical Preparations: A Systematic Review and Implications in the Indian Context

**DOI:** 10.7759/cureus.42587

**Published:** 2023-07-27

**Authors:** Veena Verma, Yashwant Lamture, Ruchira Ankar

**Affiliations:** 1 Medical Surgical Nursing, College of Nursing, All India Institute of Medical Sciences, Raipur, Raipur, IND; 2 Surgery, Acharya Vinoba Bhave Rural Hospital, Jawaharlal Nehru Medical College, Datta Meghe Institute of Higher Education & Research, Wardha, IND; 3 Medical Surgical Nursing, Smt. Radhikabai Meghe Memorial College of Nursing, Datta Meghe Institute of Higher Education & Research, Wardha, IND

**Keywords:** skin-related quality of life, chronic kidney disease-associated pruritus (ckd-ap), end-stage renal disease (esrd), chronic kidney disease (ckd), natural oil, topical preparation, uremic xerosis, uremic pruritus, topical preparations

## Abstract

Uremic xerosis and chronic kidney disease (CKD)-associated pruritus (CKD-ap) are the most commonly occurring dermatological problems faced by most of the CKD patients on hemodialysis which are not only annoying and draining to the patients but also have an intense effect on patients' quality of life. The PubMed, Scopus, Google Scholar, and Web of Science databases were searched for the literature with the following search terms: uremic xerosis OR CKD-ap OR uremic pruritus AND topical therapy OR topical ointment OR natural oil from the year 2002 -2022, and finally, 22 articles were chosen to write this review. Out of 22 studies, six used pharmacological preparations and remaining 16 studies used natural oils and components. All the articles were experimental studies (Pre/Quazi/RCT/True experimental) focusing on managing itch and xerosis associated with CKD and hemodialysis by topical application. The topical agents tried in various research studies are effective in managing itch and xerosis associated with CKD. They are safe, easy to use, and without allergic reactions. Natural oils like almond, chia seed, clove, glycerin, paraffin, and virgin coconut oil are readily available in home-care settings and can be used as a nurse-led intervention. Topical preparations for uremic xerosis and pruritus are effective, safe, and easy to apply on large body surface areas without systematic side effects. Natural oil-based topical preparations are cost-effective, safe, and easy to use.

## Introduction and background

One of the global health problems on the rise is chronic kidney disease (CKD) or end-stage renal disease (ESRD), which is linked with many debilitating and life-threatening complications like bone and minerals disease, electrolyte imbalances, cardiovascular complications, and cutaneous manifestations. People from low socioeconomic backgrounds are at the highest risk of acquiring CKD [1,2].

Uremic xerosis and pruritus are the most common cutaneous manifestations reported by most ESRD patients undergoing dialysis. Although not life-threatening, it intensely influences a patient's life quality and life expectancy [3]. Uremic pruritus (UP) or chronic kidney disease-associated pruritus (CKD-ap) is the most common dermatological problem after uremic xerosis. Uremic xerosis can be considered as the precursor of CKD-ap, which is irritating, troublesome, and draining for patients with end-stage renal disease [4,5].

The confirmation of UP necessitates careful diagnostic workups due to the wide variation in clinical symptoms. For a correct diagnosis, a thorough evaluation and elimination of itch from other skin or systemic disorders is required [6]. Around 90% of dialysis patients have reported itching, sometimes localized or generalized. The pathophysiological mechanism behind pruritus is still being determined as it has multifaceted etiological factors. Many pharmacological and non-pharmacological interventions have been researched and tried, but none are found effective in managing itch associated with CKD except kidney transplantation. There is a strong need for an evidence-based intervention for CKD-ap, especially for those not opting for transplants or waiting for surgery [7-9].

Shirazian et al. concluded in their study on 200 hemodialysis patients that CKD-ap affects the client's life quality and leads to poor sleep quality, psychological problems like irritability, depression, and overall mortality [10]. A significant correlation was reported between itch intensity, quality of life, and depressive symptoms. The study emphasizes that CKD-ap is one of the biggest and most important health problems among patients with ESRD [9].

The major causes of CKD-ap, as suggested by the multifactorial hypothesis, are an accumulation of toxic compounds, altered immune response, dysregulated opioid balance, and peripheral neuropathy. This uncertainty of causes and multifactorial pathophysiology of UP makes it more challenging for the nephrologist to decide upon the suitable protocol for treatment [11]. CKD-ap is identified as an important area of research by patients suffering from CKD. Despite this, the treating physician does not pay much attention to treating CKD-ap [12].

As expressed by CKD patients, there is a dire need for antipruritic treatment. Continuous research to develop universally acceptable, effective, and easy-to-use anti-itch treatment is under process and needs to be more conclusive. Because of UP's unclear and complex pathophysiological mechanism, the efforts are directed toward strengthening the epidermal barrier, moisturizing the skin, immune modulation, and minimizing neural response associated with itch. Topical therapy is the center piece of managing pruritus for patients unsuitable for systemic therapies [13].

Along with various topical ointments, oils from multiple plant sources remain a main line of treatment because they contain essential fatty acids and have a soothing effect on the skin. Also, it is easier to apply and cost-effective [14,15]. Sixteen small studies have examined the efficacy of various topical ointments and oil on uremic xerosis and CKD-ap, but the superiority of the treatment is not well established; hence, the optimal treatment is yet to be identified. A systematic literature review has been conducted and summarized to illuminate this critical knowledge gap and highlight the efficacy of various available topical preparations for managing uremic xerosis and CKD-ap.

## Review

Materials and methods

An exhaustive systematic review was undertaken to outline the results of all published research articles for managing uremic xerosis and pruritus with topical therapy. A scholarly search was done in the following search engines: PubMed, Google Scholar, Web of Science, and Scopus. Integrated information from all scholarly articles was presented and was internally peer-reviewed.

Search Strategy

The PubMed, Scopus, Google Scholar, and Web of Science databases were systematically searched for relevant literature using the following search terms: "Uremic xerosis" OR "CKD-ap" OR "Uremic pruritus" AND "topical therapy" OR "Topical ointment" OR "Natural oil." The inclusion criteria for this search were all research articles focusing on topical preparations for managing uremic xerosis and CKD-associated pruritus, with full-text availability in English, published until November 2022. However, studies that included patients with renal transplants or those with CKD not related to uremic xerosis or pruritus were excluded from this review. The exclusion criteria were applied to ensure that only studies relevant to managing uremic xerosis and CKD-associated pruritus were included in this review.

Article Search Process

**Figure 1:**
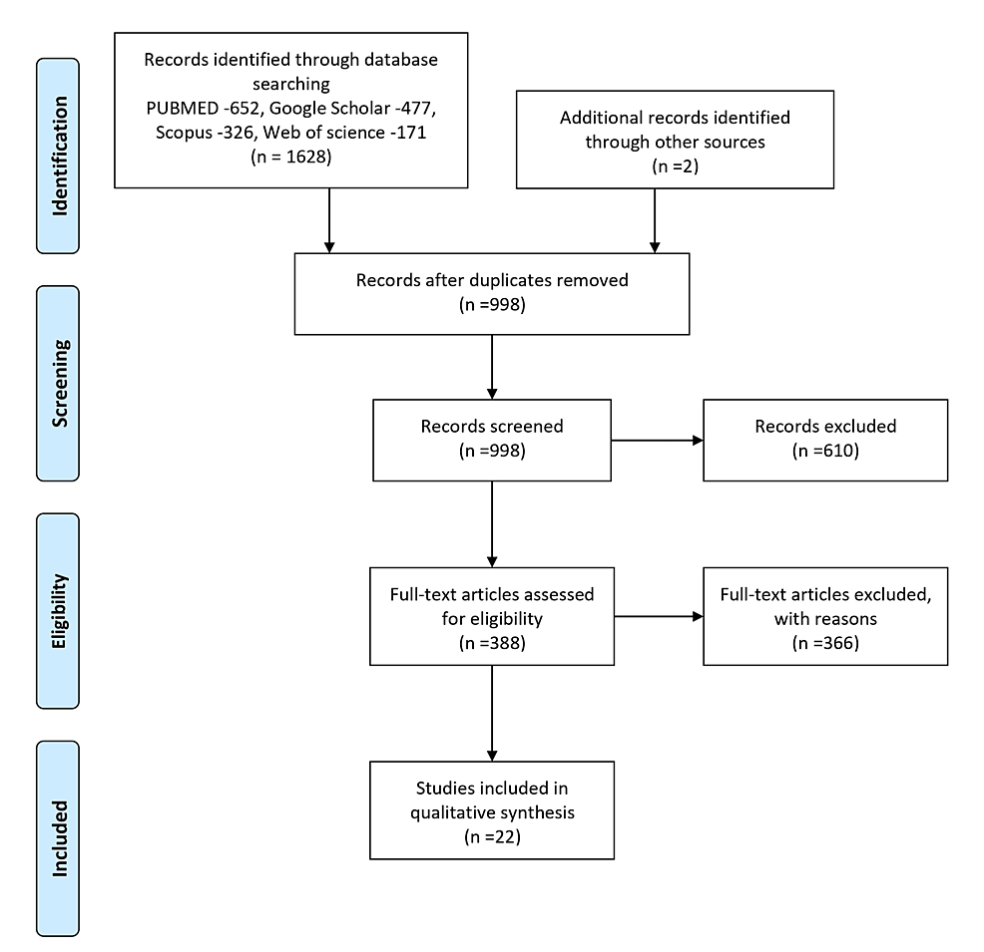
Selection process of articles used in this study Adopted from the Preferred Reporting Items for Systematic Reviews and Meta-Analyses (PRISMA).

A review based on the PRISMA (Preferred Reporting Items for Systematic Review and Meta-Analysis) guidelines was conducted to retrieve articles. The search for articles was performed on PubMed, Scopus, Google Scholar, and Web of Science databases, covering the period from 1st January 2002 to 31st December 2022. Initially, 1628 articles were identified and retrieved within this timeframe. After thoroughly examining the data and removing duplicates, only 22 articles were considered eligible for the review (as illustrated in Figure 1, demonstrating the study selection process). Of the 22 selected articles, most were experimental studies utilizing natural oils to manage Uremic xerosis and CKD-ap. The remaining six articles were focused on testing pharmacological preparations (Table 1).

**Table 1:** Details of topical preparations available for management of uremic pruritus and xerosis CKD: Chronic kidney disease; VCO: virgin coconut oil; CKD-ap: chronic kidney disease-associated pruritus

S.No	Author	Year	Intervention	Findings
1	Okada and Matsumoto [16]	2004	Aqueous gel with high water concentration	Aqueous gel with high water content should be recommended for CKD patients on hemodialysis, irrespective of itching.
2	Young et al. [17]	2009	Pramoxin-based anti-itch lotion	Pramoxine 1% lotion is safe and effective in reducing pruritus in ESRD patients on hemodialysis.
3	Balaskas et al. [18]	2011	Glycerin and paraffin	Twice daily application of emulsion containing glycerin and paraffin was found to be effective in improving the dryness and scaling of the skin, uremic pruritus and quality of life.
4	Castello and Milani [19]	2011	Topical 10% Urea + dexpanthenol lotion	Ureadin Rx 10 lotion formulation is effective in the management of uremic xerosis and pruritus.
5	Aramwit et al. [20]	2012	Sericin Cream	Sericin cream significantly decreased the pruritus and pigmentation of the skin and improved the overall quality of life among CKD patients.
6	Feily et al. [21]	2012	Cromolyn Sodium 4%	Topical preparation containing 4% cromolyn Na was more effective at reducing pruritus in 3^rd^ and 4^th^ week of its use than a placebo.
7	Lin et al. [14]	2012	Baby oil	Baby oil (cool and at normal temperature) effectively manages CKD-associated pruritus by the moisturizing and cooling effect.
8	Karadag et al. [22]	2014	Baby oil	Baby oil temporarily improved the itching and quality of sleep and life among CKD patients.
9	Nakhaee et al. [23]	2015	Avena sativa lotion and vinegar	Avena sativa lotion and Vinegar can be used as a complement to hydroxyzine therapy for managing uremic pruritus.
10	Tricaesario et.al. [24]	2016	Almond oil cream (4%)	4% Almond oil cream is a good skin moisturizer and is found to be effective in the decrease of uremic xerosis and pruritus.
11	Elsaie et al. [25]	2017	Clove oil	Clove oil is effective and more acceptable for managing uremic pruritus, especially for patients unsuitable for systemic therapy.
12	Jeong et al. [15]	2017	Chia Seed Oil	4% Chia seed oil after eight weeks of topical application improved skin hydration, licken simplus chronicus, and prurigo nodularis. Thus chia seed oil can be used to manage pruritic skin associated with CKD and other conditions.
13	Mokhtarabadi et.al. [26]	2017	Baby oil	Baby oil with temperature variation did not affect pruritus severity but is a good moisturizing agent for the skin.
14	Mehri et.al. [27]	2018	Sweet almond oil	Sweet almond oil, when applied topically, it significantly reduces uremic pruritus in CKD patients on hemodialysis patients with no complications.
15	Mehri et al. [27]	2018	Almond oil (sweet)	Sweet almond oil, when applied topically, has shown improvement in uremic pruritus.
16	Khorsand et al. [28]	2019	Violet oil	Violet oil massage is more effective in minimizing uremic pruritus than massage alone and can be a complementary therapy.
17	Aquino et al. [29]	2020	6% topical Gabapentin	Short-term use of gabapentin topically for two weeks may significantly decrease the severity of Uremic pruritus without any complications.
18	Saodah et al. [30]	2020	VCO	Virgin coconut oil improves skin hydration and is useful for managing uremic pruritus.
19	Sadeghnejad et al. [31]	2021	Ostrich oil	The ostrich oil can be used as a complementary therapy in reducing pruritus in hemodialysis patients, but no improvement in quality of life was recorded.
20	Muliani et al. [32]	2021	VCO	VCO is better than olive oil in managing uremic pruritus and can be used as a nurse-led intervention.
21	Widyastuti et al. [33]	2021	Calcipotriol 0.005% ointment	Calcipotriol ointment is more effective in managing uremic pruritus than a placebo, can be used as adjunctive treatment, and is safe for CKD-ap.

Results and discussion

Efficacy of Baby Oils in the Management of UP

Baby oil was found to effectively minimize pruritus among CKD and ESRD patients on hemodialysis in studies conducted by Lin et al. [14], Karadag et al. [22], and Mokhtarabadi et al. [26]. Variation in the temperature of baby oil was insignificant in its efficacy. One study reported that no positive correlation was found between the use of baby oil and improved quality of life [33,34].

Another study reported that topical application of baby oil improved sleep quality and positively affected the quality of life. Baby oil primarily consists of raw coconut oil and moisturizing minerals. Also, it has similarities with physiological lipids and emollients containing high water content, which is responsible for its soothing effect on the skin and itch associated with CKD-ap [16].

Effectiveness of Pramoxin-Based Anti-itch Lotion

Pramoxine, a 1% lotion, is safe and effective in reducing pruritus in CKD and ESRD patients on hemodialysis reported by Young et al. 2009 in their RCT conducted in a community setting among patients with CKD with UP. Pramoxin is a local anesthetic that relieves pain and itching by numbing the skin and thereby blocking the sensation [17].

Effectiveness of 6% Topical Gabapentin

Short-term use of gabapentin topically for two weeks may significantly decrease the severity of UP without any complications. Aquino et al., 2020, reported in their RCT (double-masked, vehicle-controlled) Gabapentin belongs to an antiepileptic drug that acts by inhibiting the influx of calcium in neurons, thereby blocking the neuropathic signals leading to itching [29].

Effect of an Emollient Containing a High Water Content on Mild UP

Based on the findings of their study, Okada et al. recommended that emollients containing a high water content should be prescribed for all CKD patients with xerosis with or without pruritus, as aqueous gel with a high water content helps manage uremic xerosis in mild pruritic CKD patients [16].

Effect and Safety of Structured Physiological Lipids' and Endo-Cannabinoids' Cream Combination for Uremic Xerosis and Pruritus

Szepietowski (2005) conducted a study to assess the efficacy of physiological lipids and endocannabinoids in managing UP among CKD patients on hemodialysis [12]. The study's findings suggest that twice daily application of ointment topically completely cured xerosis and pruritus with good tolerance. The observed reduction in pruritus may be attributed to the complete relief of skin xerosis and the endocannabinoid-induced influence on neuronal communication.

Calcipotriol 0.05% Ointment for Uremic Xerosis and CKD-ap

Widyastuti et al. (2022) conducted a double-masked, randomized trial to check the efficacy of Calcipotriol 0.05% ointment for uremic xerosis and CKD-ap [33]. Twice daily application of ointment showed significant improvement in xerosis and pruritis scores among ESRD patients on hemodialysis. Calciprotriol 0.05% minimizes keratinocyte proliferation and modulates the immune system, thereby reducing pruritus [20].

Role of Topical Avena sativa, Vinegar, and Hydroxyzine

Nakhaee et al. (2015) conducted a study to assess the effectiveness of topical preparation containing Avena sativa, vinegar, and hydroxyzine [23]. They reported that it strengthened the skin barrier and pH and moisturized the skin, hence facilitating uremic xerosis and pruritus reduction. Avena sativa is known for its skin-healing and barrier-enhancement properties, whereas vinegar maintains the acidic pH of the skin surface. Hydroxyzine has anti-allergic properties [23].

Effect of Topical Glycerol and Paraffin for Treating UP

Balaskas et al. (2011) conducted an RCT to assess the effectiveness of glycerin paraffin combination ointment among CKD patients on hemodialysis [18]. The study's findings demonstrated that topical application of products containing 10% paraffin and 15% glycerin is highly effective in treating dry skin among CKD patients, thereby also good for managing CKD-associated itch. Thus, the study emphasized that when xerosis is treated effectively, it also facilitates the irritation associated with CKD. Glycerin and paraffine are commonly used in most skin care products because they strengthen the epidermal barrier and are excellent moisturizers [22].

Effect of Sweet Almond Oil for Treating UP

Almond oil is a natural agent having emollients and an occlusive property often used to treat skin dryness and is loaded with essential fatty acids. Mehri, Afrasiabirfar, and Hosseini (2018) conducted a study to assess the efficacy of almond oil on xerosis and uremic itch. The study's findings suggest that twice daily application of sweet almond oil reduces uremic xerosis and irritation associated with uremia. Sweet almond oil is rich in linoleic acid, which moisturizes the skin along with its anti-inflammatory properties [27].

Effect of Virgin Coconut Oil in Comparison With Mineral Oil on Skin Moisture Among CKD Patients

The study done by De Las Alas et al. showed a trend toward benefit for VCO compared to mineral oil in terms of overall therapeutic response but is inconclusive since both oils improved overall dry skin scores, corner-meter readings, and patient's quality of life [34]. Hence, using either emollient may be recommended in managing uremic xerosis. A significant improvement in skin moisture was noted after topical application of VCO twice a day, as reported by Saodah et al., 2020 in their quasi-experimental study conducted on CKD patients with uremia [30]. Another study comparing VCO with almond oil said VCO is more effective than olive oil for managing uremic xerosis and pruritus [25].

Efficacy of Ostrich Oils in the Pruritus Severity and Life Quality Index in Hemodialysis Patients

Topical application of ostrich oil reduced UP but did not affect the quality of life reported by Sadeghnejad et al. Ostrich oil has anti-inflammatory action and is used widely as a massage oil to relieve tight muscles, itching, and pain [31].

Topical Violet Oil Efficacy on the Pruritus and Xerosis Severity Among Hemodialysis Patients

An RCT conducted by Khorsand et al. reported that violet oil massage is more effective than only massage in improving uremic xerosis and pruritus and can be used as a complementary therapy for patients with UP [28].

Violet oil is a plant extract of Violata Adorata (Violaceae family) found in the wild forest. From ancient times, all these plants have been used as medicinal plants. It is loaded with a range of phytochemicals like salicylic acid, methyl ester glycosides, flavonoids, saponins, vitamin C, and coumarin, responsible for its anti-inflammatory properties and skin-protecting action [27].

Topical Chia Seed Oil Efficacy on UP

Jeong et al. (2009) studied the effectiveness of 4% chia seed oil in CKD-associated pruritus, and the findings of the study suggest that chia seed significantly improved the hydration of skin along with other dermatological problems frequently noticed in CKD patients like prurigo nodularis and Licken simplex chronicus. Thus, chia seed oil can be used as adjuvant therapy for managing CKD-ap [15].

Topical Clove Oil (Eugenol) Efficacy on CKD-ap

Elsaie et al. (2017) highlighted the efficacy of topical clove oil application on symptomatic relief of pruritus associated with CKD and, based on the study findings, clove oil massage significantly reduced the uremic itch. It can be used effectively for CKD patients who are not a good candidate for systemic therapy because of poor tolerance, side effects, and contraindications [25].

Topical Cromolyn Sodium Has 4% Efficacy on Pruritus Associated With CKD

Chromolyn Na, a mast cell stabilizer, is found to be effective in managing uremic itch. A study done by Feily et al. (2013) reported that topical preparation containing 4% cromolyn Na was more effective in reducing pruritus in the third and fourth weeks of its use than a placebo [21].

Effect of Sericin Cream on Pruritus Among CKD Patients

Aramwit et al. (2012) conducted a double-masked RCT to assess the efficacy of Sericin cream on ESRD-associated itch. The study's findings suggested a significant improvement in pruritic symptoms, life quality score, and skin pigmentation with regular use of Sericin cream [20].

Sericine is the combined polypeptide chain, with serine being the most abundant amino acid, which increases skin hydration and suppresses the pro-inflammatory cytokines, thus minimizing skin irritation without any side effects [20].

Topical 10% Urea and Dexpanthenol Lotion Efficacy on Uremic Xerosis and CKD-Associated Itch

Urea is an excellent hydrating moistening agent commonly used in many dermatological preparations. Castello et al. (2011) conducted a pilot trial of topical 10% urea and dexpanthenol lotion efficacy in uremic xerosis and CKD-associated itch [19]. The study's findings suggest that it is very effective in minimizing skin dryness and irritation of uremia with excellent tolerability.

Implications in Practice

Topical therapy is the mainstay of management for uremia-associated xerosis and pruritus. Both pharmacological and natural products have been tried and used for their control. The superiority of the interventions is yet to be proved because of the lack of evidence-based data. Among the various natural topical preparations, some commonly used agents are baby oil, virgin coconut oil, and sweet almond oil are readily available in a home setting and can be initiated as nurse-lead management without any side effects.

Effective management of UP mandates the early and effective management of uremic xerosis among CKD patients even before the start of hemodialysis. The nurses working in hemodialysis areas and nephrology wards are expected to initiate skin care with natural topical components and educate the patients about its benefit over the long run.

## Conclusions

Uremic xerosis and CKD-ap are the most irking and troublesome manifestations of CKD. Topical preparations are the most common and popular ways of managing skin-related problems among CKD patients. Both natural and chemical topical preparations have effectively managed cutaneous problems among CKD patients. They act by moisturizing the skin and require no additional preparation and training on the patient’s part. The reported side effects of topical preparations are comparatively lower than the systemic drugs, especially for natural oils. Topical application and oil massage are standard routine practices among the Indian population, especially during winter, to prevent skin dryness. Thus based on the various study findings, we can conclude that the topical application of natural oils can be encouraged among CKD patients to manage uremic xerosis and CKD-ap. Nurses can utilize their every contact with patients to educate patients regarding using oils for topical applications on a routine basis.
